# Gellan-Xanthan Hydrogel Conduits with Intraluminal Electrospun Nanofibers as Physical, Chemical and Therapeutic Cues for Peripheral Nerve Repair

**DOI:** 10.3390/ijms222111555

**Published:** 2021-10-26

**Authors:** Poornima Ramburrun, Pradeep Kumar, Elias Ndobe, Yahya E. Choonara

**Affiliations:** 1 Wits Advanced Drug Delivery Platform Research Unit, Department of Pharmacy and Pharmacology, Faculty of Health Sciences, School of Therapeutic Sciences, University of the Witwatersrand, 7 York Road, Parktown, Johannesburg 2193, South Africa; poornima.ramburrun@wits.ac.za (P.R.); pradeep.kumar@wits.ac.za (P.K.); 2 Department of Plastic and Reconstructive Surgery, Faculty of Health Sciences, School of Clinical Medicine, University of the Witwatersrand, 7 York Road, Parktown, Johannesburg 2193, South Africa; elias.ndobe@wits.ac.za

**Keywords:** peripheral nerve regeneration, biocompatibility, functional recovery, nerve repair conduits, nanofibers, intraluminal guidance, nerve growth factor

## Abstract

Optimal levels of functional recovery in peripheral nerve injuries remain elusive due to the architectural complexity of the neuronal environment. Commercial nerve repair conduits lack essential guidance cues for the regenerating axons. In this study, the regenerative potential of a biosimulated nerve repair system providing three types of regenerative cues was evaluated in a 10 mm sciatic nerve-gap model over 4 weeks. A thermo-ionically crosslinked gellan-xanthan hydrogel conduit loaded with electrospun PHBV-magnesium oleate-N-acetyl-cysteine (PHBV-MgOl-NAC) nanofibers was assessed for mechanical properties, nerve growth factor (NGF) release kinetics and PC12 viability. In vivo functional recovery was based on walking track analysis, gastrocnemius muscle mass and histological analysis. As an intraluminal filler, PHBV-MgOl-NAC nanofibers improved matrix resilience, deformation and fracture of the hydrogel conduit. NGF release was sustained over 4 weeks, governed by Fickian diffusion and Case-II relaxational release for the hollow conduit and the nanofiber-loaded conduit, respectively. The intraluminal fibers supported PC12 proliferation by 49% compared to the control, preserved up to 43% muscle mass and gradually improved functional recovery. The combined elements of physical guidance (nanofibrous scaffolding), chemical cues (N-acetyl-cysteine and magnesium oleate) and therapeutic cues (NGF and diclofenac sodium) offers a promising strategy for the regeneration of severed peripheral nerves.

## 1. Introduction

The persistent challenge of adequate functional recovery in peripheral nerve injuries makes designing and investigating artificial scaffolds for nerve repair important tasks. The conventional treatment procedures, which comprise end-to-end suturing of short gap injuries (<10 mm) and autograft repair in long gap injuries (>10 mm), have several drawbacks. Direct end-to-end repair presents with increased tension across the gap defect and reduced stretching capacity of the transected nerve ends due to fibrosis, tissue adhesion and Wallerian degeneration [1]. Autograft repair is currently considered the gold standard of treatment; however, it is associated with difficulties in donor tissue harvesting, donor site morbidity and multiple surgery sites resulting in scarring. These disadvantages necessitate artificial, biodegradable and biocompatible nerve repair conduits with the goal of attaining functional recovery levels equivalent to or greater than those achieved with autograft repairs [2,3].

Despite the availability of several FDA-approved synthetic nerve repair conduits, the issues of poor mechanical strength and absence of critical guidance cues still prevail [4,5]. Peripheral nerve tissue comprises a complex cellular arrangement, with electrical, mechanical and chemical properties [6]. A multifunctional repair system, featuring a variety of specialized components, is required to emulate the native architecture and function of nerve tissue for biomimetic appeal [7,8]. The basic design of a hollow nerve conduit should provide a physical barrier from the external environment, protect from scar-tissue infiltration and retain the endogenously secreted neurotrophic growth factors, yet maintain a degree of permeability for nutrient and waste exchange [7,9]. For optimal peripheral nerve regeneration, a repair strategy should provide a biocompatible environment conducive to axonal growth, with mechanically compatible biomaterials, chemotactic and therapeutic bioactives, and physical and topographical guidance cues. The absence of physical guidance cues leads to misdirected axonal travel across long gap defects, failed or limited re-innervation of target muscles, the formation of painful neuromas and poor functional recovery [10,11]. Intraluminal physical guidance cues, in the form of gel [12] and sponge fillers [13], multiple micro-channels and aligned nanofibers [14], films and filaments, have been investigated to mimic the fascicular morphology of native nerve tissue [15,16]. Such types of intraluminal support should be incorporated at a suitable density for adequate permeation and to avoid restricting the growth of axons [14]. Chemical and therapeutic cues enhance axonal survival, proliferation and migration along the guidance scaffolds, to support regeneration [16]. The delivery of growth factors, functioning as both chemotactic and therapeutic cues, is a popular therapeutic strategy for improving the efficacy of conduits and promoting nerve regeneration [17,18]. The combined regenerative effect of a variety of cues should be investigated to provide insight into the design of an artificial repair system that closely mimics native tissue in morphology and function to improve treatment outcomes [19].

The present study investigates the in vivo nerve-regenerative potential of our previously described gellan-xanthan hydrogel conduit [20] in combination with electrospun PHBV-MgOl-NAC nanofibers as intraluminal guidance scaffolds featuring a triple guidance cue mechanism [21]. A hydrogel conduit may be able to absorb and ionically bind calcium ions entering the axoplasm, to assist with calcium-ion homeostasis and prevent calcium-induced axonal degeneration [22,23,24]. The final assembled biosimulated nerve repair device (BNRD), described herein, aims to mimic native nerve tissue for neuro-compatibility and functional recovery. The sustained delivery of NGF and DS coupled with Mg^2+^ ions, oleic acid and NAC is proposed to assist the innate repair mechanisms of nerve regeneration. The components of the BNRD system offer the following regenerative cues: (1) physical (conduit and intraluminal fibers), (2) chemical (Mg^2+^ ions, oleic acid and NAC) and (3) therapeutic (NGF and DS).

The final assembled repair conduit and its individual components were assessed for their mechanical properties and cytocompatibility before in vivo evaluations in a 10 mm gap rat sciatic nerve injury model. The effect of the different guidance cues (physical, chemical and therapeutic) on functional recovery was investigated via walking track analysis, gastrocnemius muscle mass measurement and histological analyses, as indicators of regeneration and re-innervation.

## 2. Results

### 2.1. Mechanical Properties

**Figure 1:**
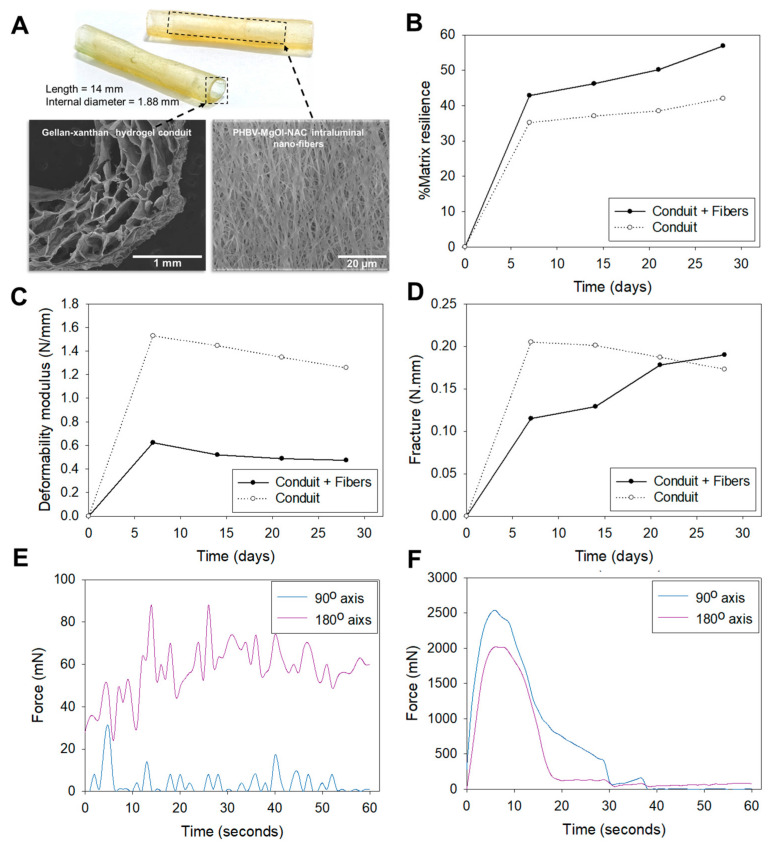
(**A**) Image of the BNRD comprising a gellan-xanthan hydrogel conduit with PHBV-MgOl-NAC intraluminal electrospun nanofibers. Mechanical profiles of hydrogel conduit over a 4 week study period conducted in PBS pH 7.4. (**B**) %matrix resilience, (**C**) deformability modulus, and (**D**) fracture. Biaxial mechanical profiles of hydrogel conduit and electrospun fibers are in dry state. (**E**) Tensile analysis of electrospun fibers and (**F**) tensile analysis of hydrogel conduit.

Textural profiling of the hydrogel conduits was conducted to assess the transition in mechanical properties induced by inserting electrospun fibers as an intraluminal filler. The conduit containing intraluminal fibers achieved a matrix resilience of 56.90% compared to 42.01% in the hollow conduit at the end of 28 days (Figure 1A). The higher matrix resilience of the fiber-loaded conduit corresponded to the lower deformability modulus of 0.47 N/mm compared to 1.26 N/mm of the hollow conduit (Figure 1B). The intraluminal fibers imparted increasing fracture energies to the conduit, whereas the hollow conduit displayed decreasing fracture energies over the 28 day study period (Figure 1C). Biaxial analysis of the hydrogel film sample and electrospun fibers showed varying tensile strengths along the 90° and 180° axes. Tensile failure of the fibrous film (Figure 1D), along the 180° axis, occurred at 14 and 26 s, with a break force of 88 mN. Multiple failure points at lower forces along the 90° axis indicated separation of individual or bundles of fibers. For the hydrogel sample (Figure 1E), increased tensile strength was noted along the 90° axis, with a break force of 2539 mN.

### 2.2. In Vitro NGF Release Profiles and Mathematical Modeling

**Figure 2:**
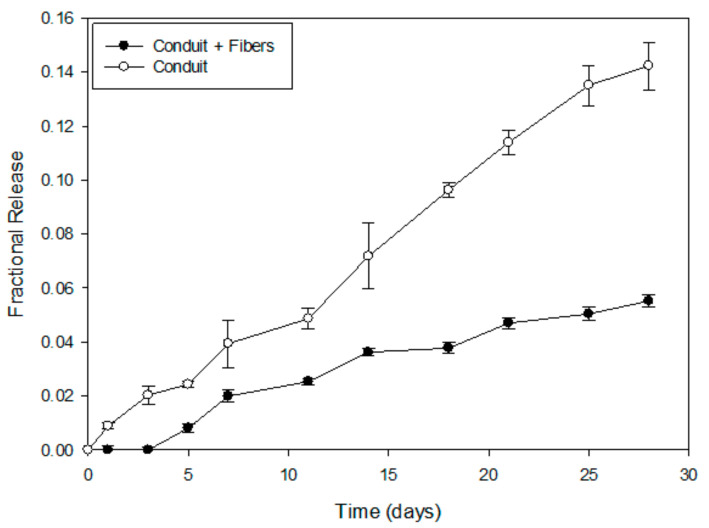
In vitro Nerve Growth Factor (NGF) release profiles from the hollow and fiber-containing conduits over 28 days in PBS pH 7.4 at 37 °C (*n* = 3).

The inclusion of PHBV-MgOl-NAC intraluminal fibers imparted a decreased overall fractional release of NGF (0.055; 11 ng) compared to the hollow conduit (0.14; 28 ng) over 4 weeks (Figure 2). Mathematical modelling (Table 1) revealed that NGF release from the conduit loaded with intraluminal fibers is best described by the Higuchi model, whereas release from the hollow conduit conforms to the Zero-order model. The diffusional exponent, *n* (Korsmeyer-Peppas equation), indicates non-Fickian anomalous transport, encompassing a coupled-diffusion and relaxation mechanism of NGF release from both the fiber-containing and hollow conduits (0.45 < *n* < 0.89). The contribution of Fickian diffusion and Case-II relaxational release of the system (R/F = K2/K1) suggest NGF release occurs via the Case-II relaxational mechanism and Fickian diffusion from the fiber-loaded conduit and hollow conduit, respectively.

### 2.3. In Vitro PC12 Proliferation

**Figure 3:**
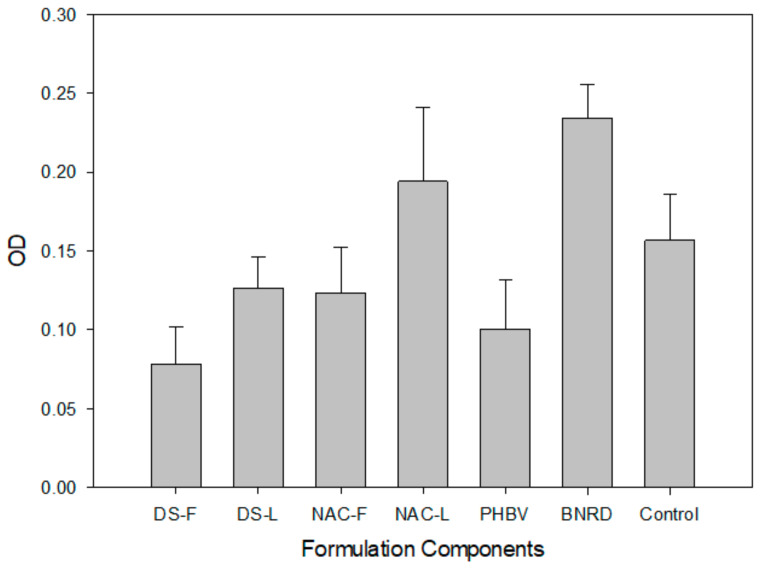
MTT PC12 viability studies in response to the drug-free (DS-F) and drug-loaded (DS-L) hydrogel conduits; the NAC-free (NAC-F) and NAC-loaded (NAC-L) electrospun intraluminal fibers; pure electrospun PHBV fibers; and the final assembled biosimulated nerve repair device (BNRD) (*n* = 3).

PC12 proliferation in response to the different scaffold components and the final assembled device was determined over 72 h (Figure 3) (*p* < 0.05). The DS-L hydrogel and the NAC-L fibers showed improved cell growth compared to the corresponding bioactive-free components (DS-F and NAC-F, respectively). The NAC-L fibers supported cell growth comparable to that of the control. Similarly, the final biosimulated nerve repair device (BNRD), comprising a DS-L conduit with NAC-L fibers, exceeded the growth of the control. The following individual components could not support the growth of PC12 cells compared to the control: DS-F, DS-L, NAC-F and pristine electrospun PHBV.

### 2.4. SEM Imaging

**Figure 4:**
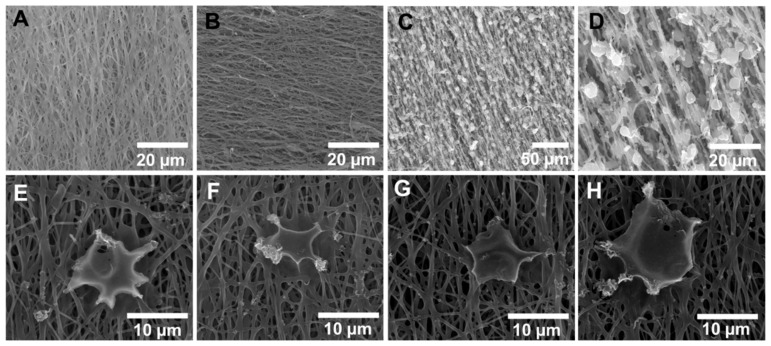
SEM imaging of electrospun fibers and PC12 cell morphology on the fibers. (**A**) NAC-loaded intraluminal fibers, (**B**) NAC-free intraluminal fibers, (**C**,**D**) pure electrospun PHBV fibers, (**E**,**F**) PC12 cell on NAC-loaded fibers, (**G**,**H**) PC12 cell on NAC-free fibers.

SEM imaging of the electrospun PHBV-MgOl fibers was used to assess surface morphology and topography of the fibers as well as PC12 cellular interactions. The NAC-loaded and NAC-free (Figure 4A,B, respectively) electrospun samples showed a random to linear deposition of smooth fibers, with an open porous network. Pure electrospun PHBV fibers depicted high linearity and a deposition of beads, which imparted an irregular and rough topography with a compact and closed structure (Figure 4C,D). The NAC-loaded fibers showed improved early neurite extension of PC12 cells (Figure 4E,F) compared to the NAC-free fibers after a 72 h incubation period (Figure 4G,F).

### 2.5. Post-Implantation Evaluation

**Figure 5:**
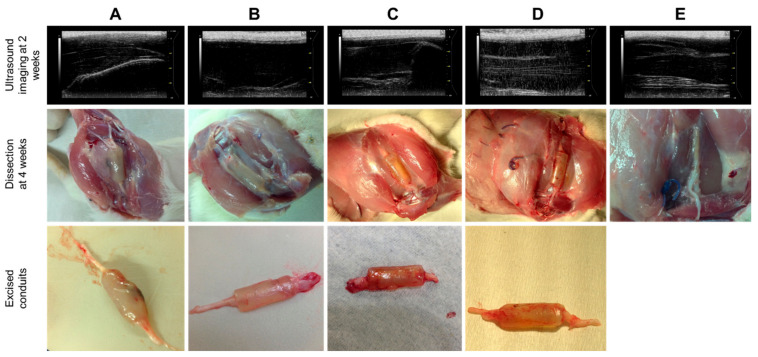
Images of ultrasound analysis, post-implantation and excised conduit of Groups (**A**–**E**).

Ultrasound imaging at 2 weeks post-surgery and dissection at the study endpoint of 4 weeks (Figure 5) revealed no significant inflammation, surgical complications or implant detachment at the operated site. The excised hydrogel conduits (Figure 5) appeared well hydrated, with a maximum wall thickness of 1.5 mm and minimal erosion at the end of 4 weeks.

### 2.6. Sciatic Function Index

**Figure 6:**
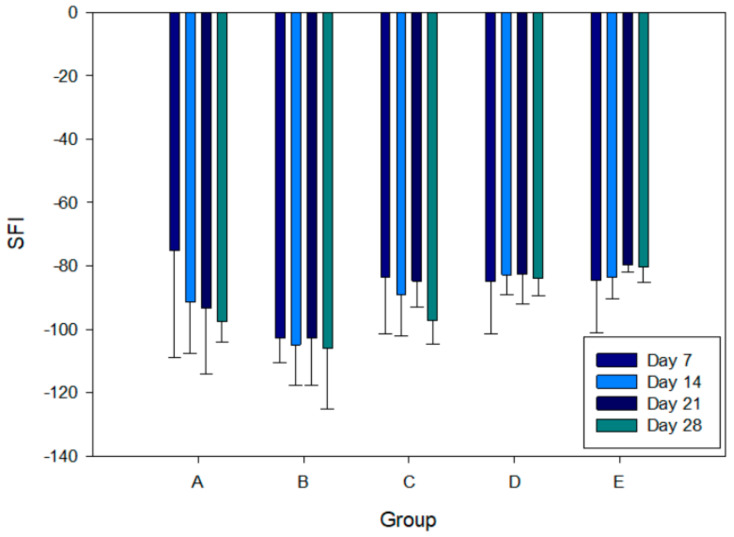
Sciatic function index determined from walking track analysis over 28 days for Groups A–E.

Sciatic Function Index (SFI) was obtained via walking track analysis as a measure of nerve regeneration and muscle innervation following functional recovery (Figure 6). The mean value of the pre-operative SFI measurement was −8.17 ± −4.15, which indicates normal nerve function. At 7 days post-surgery, the SFI values decreased substantially, indicating continued loss of nerve function. Over 7 to 28 days of the study, SFI values for the following groups progressively decreased: Group A from −91.13 ± 12.19 to −97.66 ± 6.47; Group B from −102.70 ± 7.67 to −106.12 ± 18.94; and Group C from −83.52 ± 17.76 to −97.32 ± 7.44. The SFI values for Group D remained constant between −84.95 ± 16.62 and −84.02 ± 5.34, and those of Group E showed slight improvements from −84.54 ± 16.57 to −80.17 ± 4.96, over the course of the study. Group B performed the poorest and obtained the lowest SFI of −106.12, which indicated complete nerve dysfunction and statistical significance (*p* < 0.05). Compared to Group B, Group C received an identical but DS-NGF-loaded conduit and exhibited improved function (SFI of −97.32 at 28 days); however, this was statistically insignificant compared to the other groups (*p* > 0.05).

### 2.7. Muscle Mass and Histology

**Figure 7:**
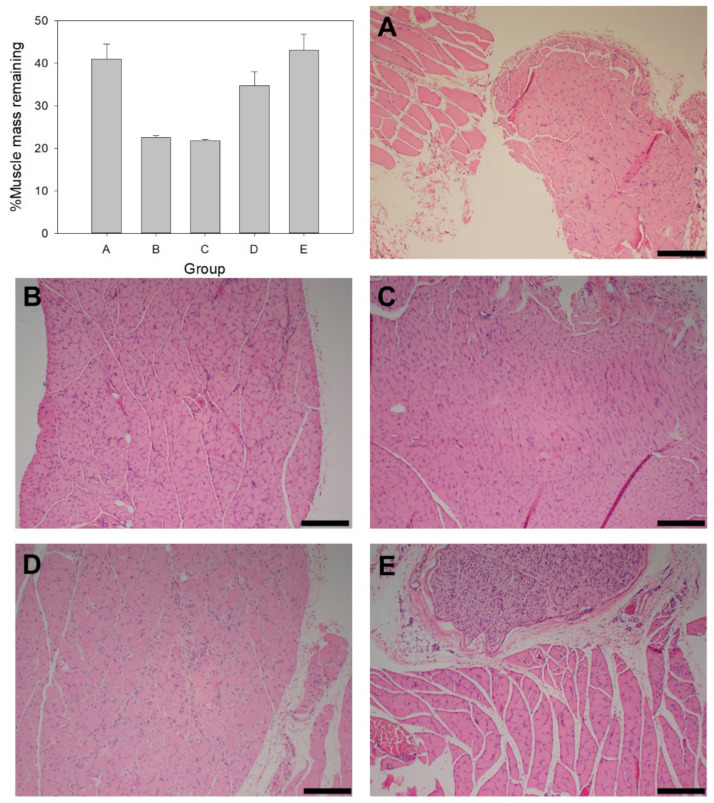
%Muscle mass remaining and histological images of gastrocnemius muscle tissue at 28 days in Groups (**A**–**E**) (Scale bar= 200 µm).

The degree of nerve regeneration and muscle reinnervation was assessed via %muscle mass remaining and histology of the gastrocnemius muscle. At the end of 4 weeks, the %muscle mass remaining for each of the groups was as follows: Group A 40.96 ± 3.52%, Group B 22.44 ± 0.53%, Group C 21.71 ± 0.20%, Group D 34.74 ± 3.26%, and Group E 43.05 ± 3.75%. Compared to the others, Group C and E were statistically significant (*p* < 0.05). In addition to similar muscle mass, histology showed that Group B and C had similar muscle tissue morphology of reduced muscle fiber size and deposition of connective tissue between muscle bundles (Figure 7B,C, respectively). Group A and E (Figure 7A,E, respectively) displayed increased muscle interstitial cell nuclei, reduced muscle fiber shrinkage and minimal connective tissue between muscle bundles, thus indicating healthier muscle tissue compared to Groups B, C and D (Figure 7).

### 2.8. Nerve Histology

**Figure 8:**
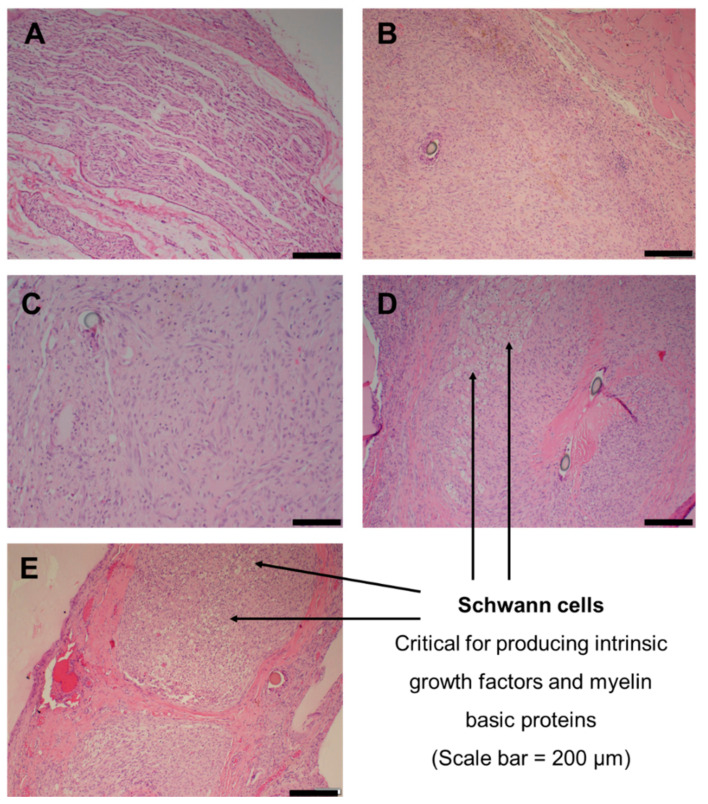
Histological images of regenerated sciatic nerve tissue in Groups (**A**–**E**) at 28 days.

Histological analysis on the regenerated nerve segment at the mid-portion of the conduit at 28 days was performed to assess the degree of nerve regeneration (Figure 8). All specimens collected from Groups A–E demonstrated a mild inflammatory response. Group A presented with normal peripheral nerve tissue, increased cellularity from spindle and Schwann cell proliferation (Figure 8A). Karyopyknosis, an event leading to nuclear apoptosis, was noted. Group B showed axonal damage, the increased presence of spindle cells and granulomatous inflammation, which obscured the underlying peripheral nerve tissue; however, an interlaced formation of spindle cells represented reactive Schwann cells (Figure 8B). Inflammation consisting of lymphocytes, plasma cells and mast cells was present along the interstitium. Group C presented with extensive proliferation of interstitial cells and granulomatous inflammation consisting of epitheloid macrophages (Figure 8C). Group D presented with spindle cells, numerous capillaries, red blood cells, fibrin deposition and Schwann cells (Figure 8D), with granulomatous inflammation. Group E exhibited moderate interstitial cells, Schwann cell proliferation and neovascularization, accompanied by inflammation that consisted of lymphocytes, plasma cells and epitheloid macrophages (Figure 8E).

## 3. Discussion

### 3.1. Mechanical Properties

Porosity within a polymer matrix imparts the property of resilience, which allows the matrix to undergo plastic deformation upon compression. Singular and large unoccupied voids, such as the hollow lumen of the hydrogel conduit, may potentiate a decrease in resilience due to compromised strength against compressive stress. Incorporating the electrospun fibers in a spiral configuration within the conduit lumen improved resilience (Figure 1A), as it partially occupied the large void of the hollow lumen. Compressive forces may be exerted by the surrounding muscle tissues during in vivo implantation, and enhanced resilience ensures the conduit’s architecture is maintained without permanent collapse or deformation [25]. Corresponding to improved resilience is decreased deformation and increased fracture energy (Figure 1C,D respectively) of the conduit with intraluminal fibers, which indicates the shift from a rigid towards a more flexible scaffold. The layers of the coiled electrospun film assist the conduit to deform and dissipate energy from an applied force to undergo a temporary change in shape rather than resist the energy, which eventually leads to fracture of the scaffold. This confers the conduit with the biomechanical compatibility to withstand forces generated from body movements and to conform to the tissue shape rather than resist deformation, which creates the potential for discomfort and pain. The post-implantation images (Figure 5) show a well-maintained structure of the conduit before and after excision, which demonstrates the adequate structural integrity of conduit throughout its in vivo implantation.

The difference in tensile strength along the 90° and 180° axes is imparted by weak inter-fiber adhesion points associated with the pattern of fiber deposition and orientation during the electrospinning process (Figure 4A,B). A tensile force applied perpendicular to the fiber orientation results in the separation of individual or small groups of fibers, whereas force applied parallel to the fiber orientation results in slight stretching of the fibers prior to onset of mechanical failure. A similar observation for the hydrogel sample could be due to the molecular arrangement of the polymer chains in a specific direction during the crosslinking, molding and drying processes. The tensile strength along both axes was sufficient to withstand elastic retraction of the severed nerves and tensile strain from the sutures, which connected the conduit ends to the nerve epineurium.

### 3.2. Drug Release Kinetics of NGF

The delivery of neurotrophic growth factors, such as NGF, is crucial during the early phase of injury for neuronal survival, growth promotion and differentiation of the regenerating axons [26]. Several studies have shown that the release of high doses of NGF may hinder nerve regrowth due to down-regulation of TrkA receptors in the presence of excessive NGF molecules [27,28]. Depending on the extent of peripheral nerve injury, lower doses of exogenous NGF may be suitable, as the neuronal tissue is capable of synthesizing endogenous NGF preceding Wallerian degeneration. In this case, the objective would be to assist the body to repair itself rather than coerce the repair process. Therefore, studying the release profiles of neurotrophic growth factors from a repair scaffold is important to determine the release kinetics, the mechanisms that govern release and the final dose delivered to the target tissues.

The 2.5-fold decrease in NGF release and its absence at days 1 and 3, resulting from the inclusion of intraluminal fibers, could be attributed to the entrapment of NFG molecules within the fibrous network upon its escape from the crosslinked hydrogel conduit. PHBV is an anionic polymer, whereas NGF, at pH 7.4, carries a positive charge; this potentiates ionic adhesion to the polymer chains. The Higuchi model, ascribed to NGF release from the conduit with intraluminal fibers, indicates the role of diffusion as a release mechanism of the peptide from the hydrogel conduit to the PHBV-fibrous network [29,30]. Polymer relaxation following erosion would be required for the peptide’s subsequent release from the fibrous network; hence, the Case-II relaxational mechanism was determined from kinetic modelling [30,31,32]. This type of delayed release and entrapment of NGF within the intraluminal guidance fibers could be favorable, as it decreases NGF wastage, its excessive leakage out of the conduit system, and the potential downregulation of TrkA receptors, leading to hindered axon regeneration. In this way, the released NGF molecules would be confined to the interior of the conduit lumen, where they would be readily available to the regenerating axons, as opposed to escape outside the conduit. Attached molecules of NGF on the surface of the PHBV fibers may enhance neuronal cell interaction and adhesion by improving the physical guidance ability of the fibers towards innervation of the target muscles. This effect functions as both a chemical cue and a therapeutic cue.

### 3.3. In Vitro Cytocompatibility

The enhanced growth of PC12 cells observed for the PHBV electrospun fibers containing both MgOl and NAC reflects the favorable effects of these constituents. The presence of MgOl improved the compatibility of pristine PHBV fibers (Figure 3, NAC-L vs. PHBV), whereas NAC considerably boosted cell proliferation. The reduced cell viability noted for DS-F and PHBV could be attributed to the degradation of carboxylic acid moieties in PHBV (butyric and valeric acids), gellan gum and xanthan gum (glucuronic and pyruvate acidic groups in both gums), which decrease the microenvironmental pH to induce cellular stress. The uneven textured and bead-like topography of the pristine PHBV electrospun fibers (Figure 4C,D) could further reduce cell compatibility and interaction. Similarly, DS exhibited potential as a neuronal protectant, as it improved cell viability of the gellan-xanthan conduit (Figure 3, DS-F vs. DS-L). The increased cell proliferation achieved with the BNRD system suggests that combinations of DS, MgOl (magnesium ions and oleic acid) and NAC could function coactively to improve neuronal survival and growth via neuro-protective actions [33]. Improved neuronal survival translates to a greater percentage of viable cells in a stressed microenvironment. There is a possibility that oleic acid could offer some neurotrophic benefit in peripheral nerve injuries, similar to axonal development in brain tissue via increasing expression of axonal growth-associated protein and microtubule-associated protein-2 [34,35,36]. In addition to preventing cellular oxidative damage, NAC acts as a neuroprotectant against cell death associated with insufficient availability of trophic factors [37,38]. The potential of the intraluminal fibers to provide a physical guidance cue and that of MgOl and NAC to function as chemical cues is noted during early neurite extension along the fibrous network (Figure 4G,H). The effects of NAC may promote neurite extension, with the possibility of encouraged cellular adhesion to the fibrous substrate [39]. The improved cell viability and biocompatibility displayed in response to the final BNRD system confirmed safety for further investigation of this system in the rat in vivo model.

### 3.4. Functional Recovery Evaluation

The degree of muscle reinnervation and return of motor function was assessed based on the SFI measurements (Figure 6). Comparing Groups C to A and B, the improved functional recovery and the potential reparative role of the anti-inflammatory DS [40] and neurotrophic NGF in the treatment of peripheral nerve injuries was noted. Despite the slight positive effect noted in this study, the benefit of DS and other nonsteroidal anti-inflammatory agents for peripheral nerve regeneration and neuroprotection requires further dose-dependent mechanistic studies [41]. Groups D and E, compared to the other groups, demonstrated improved functional recovery, attributed to the intraluminal fibers as a physical guidance cue. The neuro-protective effect of NAC in the electrospun fibers is shown in the enhanced functional recovery of Group E compared to D. These results suggest that MgOl and NAC were effective in protecting and promoting neuronal growth, which was consistent with the MTT proliferation assay. In addition to this, the intraluminal fibers provided physical and topographical guidance to support axonal growth towards the target muscle for reinnervation. The NGF loading dose of 100 ng and its release kinetics could be considered adequate for nerve injury treatment and regeneration.

### 3.5. Gastrocnemius Muscle Mass and Histology

Muscle mass has been utilized as an effective indicator of recovery and reinnervation after peripheral nerve injury. As a result of immobility, de-innervated muscles begin to atrophy, leading to decreased muscle fiber size and overall muscle mass. Increased gastrocnemius muscle mass suggests improved re-innervation by the regenerating axons of the sciatic nerve, thereby reducing the extent of muscular atrophy. The effects of DS, MgOl and NAC promoted muscle fiber health, which was indicated by the presence of numerous interstitial cell nuclei and overall muscle fiber size [42,43,44]. The PHBV-MgOl-NAC intraluminal guidance fibers improved %muscle mass (Figure 7) in Groups D and E compared to the hollow hydrogel conduit in Groups B and C (which presented with the lowest %muscle mass). This indicates the importance of physical and topographical guidance cues in achieving improved levels of muscle re-innervation. SFI measurements and muscle mass in Groups D and E suggest enhanced axonal guidance, resulting in improved target muscle re-innervation and functional recovery, comparable to that of the autograft repair in Group A.

### 3.6. Sciatic Nerve Regeneration and Histology

Nerve histology of the regenerated tissue segments provided additional insight into the efficacy of the different cues implemented: physical, chemical and therapeutic. Mild inflammation observed across the five groups was attributed to the presence of foreign material, such as the sutures, the conduit and the guidance fibers. Although granulomatous inflammation was evident, the absence of excessive fibrotic tissue may suggest the role of DS as an anti-inflammatory agent in Groups C, D and E. The reversed nerve autograft presented with normal peripheral nerve tissue and Schwann cells, since its cellular integrity was conserved, as evidenced by the %mass muscle mass maintained. Peripheral nerve regeneration requires Schwann cell proliferation to produce intrinsic neurotrophic factors and myelin basic protein, which are crucial for the effective regeneration of damaged nerve tissue [45]. The prominent appearance of Schwann cells and blood vessels in Groups D and E, compared to Groups B and C, further supports the notion of MgOl and NAC as neuroprotectants for the survival and growth of sprouting axons. The lack of physical guidance in Groups B and C may have contributed to the formation of bundled misdirected axons and hence the presence of granulomatous inflammation and the very low remaining %muscle mass.

## 4. Materials and Methods

### 4.1. Materials

Gellan gum (low acyl GelzanTM CM), xanthan gum, poly(3-hydroxybutyric acid-co-3-hydroxyvaleric acid) (PHBV) (PHV content 12 mol%) natural origin, diclofenac sodium, N-acetyl-L-cysteine (NAC) (≥99% TLC), 2,2,2-trifluoroethanol (≥99% GC) and dialysis tubing cellulose membrane (molecular weight cut-off = 14,000 Da) were purchased from Sigma Aldrich, Steinham, Germany. Polymethyl methacrylate (PMMA) (Eudragit S100) was purchased from Evonik, Midrand, Johannesburg, South Africa, and was used without further modifications. Propylene glycol (PG), sodium hydroxide pellets, chloroform, oleic acid vegetable (extra pure) and Span 80 were purchased from Merck, Darmstadt, Germany, and calcium chloride (CaCl_2_) anhydrous was purchased from Rochelle Chemicals, Johannesburg, South Africa. Magnesium sulphate heptahydrate CP (MgSO_4_·7H_2_O), methanol, acetone and isopropyl alcohol were purchased from Associated Chemical Enterprises, Southdale, Johannesburg, South Africa. Ethanol (99% absolute) was purchased from LabChem, Edenvale, Johannesburg, South Africa. PeproTech^®^ recombinant human β-nerve growth factor (NGF) and corresponding ELISA kits were sourced from Celtic Molecular Diagnostics, Cape Town, South Africa. Milipore water was used for washing of preparations. Dulbecco’s Modified Eagle Medium (DMEM), Donor Equine Serum (DES) and Fetal Bovine Serum (FBS) were procured from Hyclone, Separations, South Africa. Penicillin/Streptomycin/Amphotericin B (P/S/AB) solution and the Roche Cell Proliferation Kit I were purchased from Lonza, Morristown, NJ, USA, and Sigma-Aldrich, St. Louis, MO, USA, respectively.

### 4.2. Synthesis of the Biosimulated Nerve Repair Conduit

A 1% *w*/*v* gel solution, in 100 °C deionized water, was prepared from a 70:30 ratio of gellan gum and xanthan gum powders. Added to this solution were propylene glycol (5% *w*/*v*) as a plasticizer, calcium chloride (0.05 *w*/*v*) as a crosslinker, DS (0.4% *w*/*v*) as a bioactive, and PMMA (25% *w*/*w*) as pH-responsive particles. The gel solution was poured into molds, dried and then cut into 14 mm length conduits. Nerve growth factor was loaded by micro-pipetting 20 uL (200 ng) of NGF solution into the center of the conduit to absorb through and dry in a sterilized fumehood for 8 h. Magnesium oleate (MgOl) was synthesized using oleic acid and MgSO_4_.7H_2_O, as previously reported [18,46]. A 7.5% *w*/*v* solution of PHBV (in a 3:2 mixture of chloroform and trifluoroethanol at 60 °C) with 35% *w*/*w* of MgOl and 2% *w*/*v* NAC was electrospun into fibrous films (NanoSpinner24, Inovenso, Turkey) using a rotating drum at 1200 rpm, with a voltage of 25 kV, flowrate of 2 mL/h and nozzle distance of 150 mm. The electrospun films (10 µm thickness) were cut into 10 mm × 20 mm lengths, folded in half to form 10 mm × 10 mm square films, and then rolled into a tight spiral and inserted into the lumen of the hollow conduits. For improved visualization and mechanical stability during surgical implantation, the conduit ends were coated with a 10% *w*/*v* solution of PMMA in isopropyl alcohol.

### 4.3. Assessment of Mechanical Properties

A texture analyzer (TAXTplus Stable Microsystems, Surrey, UK) fitted with a 10 mm diameter delrin probe, set to a pre-test and post-test speed of 1.00 mm/s, a test speed of 0.5 mm/s, a trigger force of 0.5 N and a load cell of 5 kg with a target mode of 10% strain, was used to assess the matrix resilience, deformability modulus and fracture energy of conduits hydrated in phosphate buffered saline (PBS) pH 7.4 at pre-determined time points. Biaxial tensile strength of the conduit and the PHBV-MgOl-NAC electrospun films were assessed at a strain magnitude of 10% and 100%, respectively, for 60 s using a 250 mN load cell (BioTester5000 Biaxial Test System, CellScale, Waterloo Instruments Inc., Waterloo, ON, Canada).

### 4.4. In Vitro NGF Release Study

Release studies were performed over 28 days using 10 mm conduits loaded with 200 ng of NGF. Samples (*n* = 3) were placed in glass vials with 1 mL PBS pH 7.4 and stored in an orbital shaking incubator at 37 °C and 25 rpm/minute. At pre-determined time intervals, 0.5 mL of dissolution media was removed and replaced with an equal fresh volume to maintain sink conditions. The quantity of NGF released was determined using an NGF-ELISA kit, and the drug-release mechanisms were obtained via kinetic modelling using the Zero-order, First-order, Higuchi, Hixson-Crowell, Hopfenberg, Korsmeyer-Peppas and Peppas-Sahlin models.

### 4.5. In Vitro Cell Proliferation Assessment

The rat adrenal gland pheochromocytoma PC12 mixed adherent/suspension cell line (CPC-12C) obtained from Cellonex, Separations, South Africa, was cultured in a humid 5% CO_2_ atmosphere at 37 °C. The culture medium (replaced every 2 days) comprised DMEM with 10% *v*/*v* DES, 5% *v*/*v* FBS and 1% *v*/*v* P/S/AB solution. Cell proliferation in response to the various components of the nerve repair system was assessed using the MTT proliferation assay. Samples of 10 mm × 10 mm were sterilized under UV light for 12 h, incubated overnight in 400 μL of culture medium in a 48-well plate, and then seeded with PC12 cells at a density of 2 × 10^4^ cells/well and incubated for 72 h. Studies were performed in triplicate (*n* = 3) using the following samples: diclofenac-free (DS-F) and diclofenac-loaded (DS-L) hydrogel conduits, NAC-free (NAC-F) and NAC-loaded (NAC-L) electrospun films, pristine PHBV electrospun films, and the final assembled device (DS-L conduit + NAC-L fibers).

### 4.6. Visualization of Fiber and Cell Morphology

Visualization of surface morphology of the electrospun films and of the seeded cells was conducted via scanning electron microscopy (SEM) (FEI Nova NanoLab 600TM, FEI Company, Hillsboro, OR, USA). Samples were fixed to aluminum stubs using double-sided adhesive carbon tape and then sputter-coated with carbon and gold-palladium for 120 s (SPI ModuleTM Sputter-Coater and Control Unit, West Chester, PA, USA). The samples were further analyzed for elemental composition using SEM-energy dispersive X-ray spectroscopy (EDS). Cell-seeded samples were washed 3 times with PBS 1×, frozen at −80 °C for 24 h, and then freeze-dried before sputter coating.

### 4.7. Animal Study

Sprague Dawley rats, male and female (250–300 g), were procured from the Central Animal Services (CAS), with ethics clearance (2014/42/C) from the Animal Ethics Screening Committee of the University of the Witwatersrand. All surgical procedures and peri-operative care were executed in accordance with the standard protocols of the CAS, the South African Standard for the use and care of animals for scientific purposes, and the National Institutes of Health Guide for Care and Use of Laboratory Animals. The study consisted of 5 treatment groups in a 10 mm sciatic nerve gap defect (*n* = 6) (Table 2), and the conduits were sterilized via exposure to UV light for 18 h prior to surgery.

### 4.8. Surgical Procedures

Rats were anesthetized with an intraperitoneal mixture of ketamine (100 mg/kg) and xyalazine (5 mg/kg). Anesthesia was maintained during the surgery with 1.5% isoflurane in oxygen. The left thigh and hind limb of the rats were shaved and disinfected. A 2–3 cm incision was made along the thigh, and the gluteus maximus muscle was retracted to expose the left sciatic nerve. A 5 mm segment of nerve was excised at the mid-thigh level, proximal to the tibial and peroneal bifurcation, to create a 10 mm gap. The nerve gap was bridged either by a 14 mm conduit at a depth of 2 mm or a 180° reversed nerve autograft using 2 single epineurial 9/0 polypropylene sutures on either end. The muscles and fascia layers were closed with running 4/0 vicryl absorbable sutures, and the skin was closed with single 4/0 non-absorbable nylon sutures. Post-operative pain was managed with injections of buprenorphine of 0.05 mg/kg for 3 days. At the 4 week endpoint of the study, the animals were euthanized with 100 mg/kg pentobarbital administered intraperitoneally.

### 4.9. Walking Track Analysis

Walking track analysis was conducted weekly to assess functional recovery in terms of sciatic function index (SFI). The following measurements were taken for the experimental left hind limb (E) and the normal right hind limb (N): the lengths between the third toe and the heel: print length (PL); the first toe and the fifth toe: toe spread (TS); the second toe and the fourth toe: intermediary toe spread (IT). The SFI was calculated using the formula: SFI = −38.3 × (EPL − NPL)/NPL + 109.5 × (ETS − NTS)/NTS + 13.3 × (EIT − NIT)/NIT − 8.8 [47,48]. A SFI value approaching −100 indicates nerve dysfunction; therefore, increasing SFI values indicate improved functional recovery.

### 4.10. High-Frequency Ultrasound Imaging

Ultrasound imaging was conducted at 14 and 28 days to visualize abnormalities, such as detachment of the conduit or mass inflammation, post-surgery. The rats were restrained on a heated stage and maintained under anesthesia with 1.5% isoflurane in oxygen. The ultrasound machine was equipped with a probe of 40 MHz and 6 mm focal depth and had a spatial resolution of 40 × 80 × 80 μm^3^ (Vevo 2100 High-Frequency Ultrasound System, Visual Sonics, Inc., Toronto, ON, Canada).

### 4.11. Gastrocnemius Muscle Mass Measurement

Muscle mass of the gastrocnemius muscles was assessed at the endpoint of the study at 28 days. The gastrocnemius muscle from both the left and right sides was dissected out immediately after euthanizing the animals with 100 mg/kg pentobarbital, administered intraperitoneally. The muscles were weighed while wet, using an electronic balance. The percentage ratio of the remaining muscle mass was calculated as a measure of functional recovery and re-innervation.

### 4.12. Muscle and Nerve Histological Assessment

The gastrocnemius muscle and the regenerated nerve tissue were collected at 28 days post-implantation for histological analysis. The nerve sections, collected at the midpoint of the conduit, were assessed for signs of Schwann cell proliferation and regeneration, whereas the gastrocnemius muscles were assessed for signs of atrophy. The tissues were stored in a 2.5% glutaraldehyde and 0.1 M PBS solution for 2 h before being fixed in 1% osmium tetraoxide for 1.5 h. The specimens were washed with PBS, dehydrated with alcohol, embedded in wax blocks and then sectioned into 5–6 μm samples using a microtome. The cross-sections were stained with haematoxylin and eosin and viewed under a light microscope.

### 4.13. Statistical Analysis

All data were expressed as mean values ± standard deviation. Statistical analysis was performed using the Student *t*-test and a statistical significance of *p* < 0.05.

## 5. Conclusions

Mechanical profiling, PC12 proliferation assays and NGF release kinetics showed adequate in vitro biocompatibility and durability of a biosimulated nerve repair device comprising a gellan-xanthan hydrogel conduit and PHBV intraluminal nano-fibrous guidance cues. Assessment of in vivo physical recovery, derived from SFI measurements, suggests the gradual return of functional recovery and motor control in the groups implanted with the fully assembled BNRD system. Morphometric analysis of the gastrocnemius muscle mass showed reduced muscle atrophy in the groups treated with the PHBV-MgOl (Group D) and PHBV-MgOl-NAC (Group E) intraluminal guidance cues compared to those treated with only the hollow hydrogel conduits. Histological analysis of the regenerated nerve segments indicated Schwann cell proliferation, neural-type cellular arrangement and neovascularization in the former groups. These observations suggest that implementing a trio of physical, chemical and therapeutic guidance cues may simulate a neuronal microenvironment conducive of axonal repair, particularly in the early stages of nerve regeneration. Results from this study may benefit prospective designs of nerve repair conduits; the hydrogel conduit and electrospun fibers studied herein hold promise for further investigations over lengthened post-operative durations (≥6 weeks) and in long gap (≥10 mm) peripheral nerve injuries.

## Figures and Tables

**Table 1:** Kinetic data of NGF release.

Mathematical Model	Parameter	Conduit	Conduit + Fibers
Zero-Order	R^2^	0.907	0.960
First-Order	R^2^	0.904	0.962
Higuchi	R^2^	0.884	0.975
Hopfenberg	R^2^	0.905	0.961
Hixson–Crowell	R^2^	0.905	0.961
Korsmeyer–Peppas	R^2^ *n*	0.958 0.850	0.821 0.622
Peppas–Sahlin	R^2^ K_1_ K_2_ R/F Ratio	0.907 262.240 518.640 1.978	0.828 185.630 5034.400 27.121
**Best-fit model**		Zero-Order	Higuchi

**Table 2:** Animal groups and type of treatment received over 4 weeks (*n* = 6).

Group	Treatment	Bioactive Dose
A	Reversed autograft	-
B	Bioactive-free conduit	-
C	Bioactive conduit	DS = 4 mg NGF = 100 ng
D	Bioactive conduit + bioactive-free intraluminal fibers	DS = 4 mg NGF = 100 ng
E	Bioactive conduit + bioactive intraluminal fibers	DS = 4 mg NGF = 100 ng NAC = 0.7 mg

## Data Availability

The raw data presented in this study are not publicly available but may be available for researchers on request from the corresponding author after a special review that includes approval of the research project.
